# The prognostic value of the HALP score among patients diagnosed with Hodgkin lymphoma

**DOI:** 10.1097/MD.0000000000051347

**Published:** 2026-10-30

**Authors:** Mesut Ayer, Gunhan Tekin, Edibe Sevde Eker, Istemi Serin

**Affiliations:** a Department of Hematology, University of Health Sciences, Istanbul Basaksehir Cam and Sakura City Hospital, Istanbul, Turkey; b Department of Hematology, University of Health Sciences, Tepecik Training and Research Hospital, Turkey.

**Keywords:** efficacy, HALP score, Hodgkin lymphoma, prognosis, survival

## Abstract

The hemoglobin, albumin, lymphocyte, and platelet (HALP) score is an immune-nutritional biomarker utilized for prognostic assessment in oncology patients. It is determined by the formula: hemoglobin (g/L) × albumin (g/L) × lymphocytes (×10^9^/L)]/platelets (×10^9^/L). The objective of this study is to evaluate the prognostic value of the HALP score and its association with treatment response and survival outcomes among patients diagnosed with Hodgkin lymphoma (HL). Patients diagnosed with HL at the Hematology Clinic of Istanbul Başakşehir Çam and Sakura City Hospital between January 1, 2020, and January 1, 2025, were included in this study. A total of 100 patients with HL were included in the study. Patients were categorized based on the median HALP score of 23.72. Patients with a low HALP score exhibited significantly higher rates of stage IV disease, presence of B symptoms, and bulky masses. Patients with a high HALP score demonstrated significantly more complete response and less progression at the end of treatment. The analysis revealed a statistically significant 2.82-fold (95% confidence intervals: 1.14–6.97) increased risk of progression in patients with a HALP score of <23.72 compared to those with a score of ≥23.72. Patients with lower HALP scores exhibited more adverse disease characteristics. Lower HALP scores were associated with adverse disease characteristics, poorer treatment response, and shorter progression-free survival; however, HALP did not remain an independent prognostic factor in multivariate analysis.

## 1. Introduction

Hodgkin lymphoma (HL) is a hematologic malignancy characterized by a bimodal distribution, affecting both young adults and individuals aged 55 years and older.^[1,2]^ Recent medical advancements and the development of novel treatment modalities have altered the prognosis, notably enhancing the likelihood of cure, particularly with the introduction of immune checkpoint inhibitors.^[1-6]^

Various risk factors have been identified to predict clinical outcomes in addition to the HL staging system. Risk factors for early-stage HL include the presence of B symptoms, a large mediastinal mass or bulky mass, an increased sedimentation rate, multiple nodal involvements, extranodal involvement, and being 50 years of age or older.^[7]^ In advanced-stage HL, the presence of poor prognostic indicators, such as albumin levels below 4 g/dL, hemoglobin levels below 10.5 g/dL, age >45 years, stage IV disease, and the presence of leukocytosis or lymphopenia, is indicative of a more unfavorable prognosis.^[8]^ Metabolic tumor volume, assessed through pretreatment positron emission tomography (PET) and computed tomography scans (PET-CT), also aims to estimate tumor burden and risk.^[9,10]^

The hemoglobin, albumin, lymphocyte, and platelet (HALP) score is an immune-nutritional biomarker utilized for prognostic assessment in oncology patients, initially identified in individuals with gastric cancer.^[11]^ The HALP score is determined by the formula: hemoglobin × albumin × lymphocytes/platelets. Patients exhibiting high HALP scores demonstrated improved prognoses and extended survival durations.^[11]^ The potential efficacy of the treatment has been noted beyond solid tumors, including in lymphoma. Research findings indicate that patients diagnosed with diffuse large B-cell lymphoma and possessing low HALP scores exhibit reduced 3-year overall survival compared to those with high HALP scores.^[12]^ While the role of the HALP score in hematological malignancies remains inadequately defined, it represents a promising area for further investigation. Given its simplicity in application and calculation, as well as its potential to accurately reflect a patient’s immune and nutritional status, the primary aim is to expand upon the existing literature and contribute to the prognostic understanding of HL. The objective of this study is to evaluate the prognostic value of the HALP score and its association with treatment response and survival outcomes among patients diagnosed with HL.

## 2. Materials and methods

This study is a retrospective analysis including patients diagnosed with classical HL between January 2020 and January 2025 at the Hematology Clinic of Başakşehir Çam and Sakura City Hospital. The inclusion criteria were as follows: patients had histopathologically confirmed classical HL; patients for whom initial diagnostic information, treatment details, and follow-up data are both accessible and complete; patients aged ≥18 years. The exclusion criteria were as follows: patients had concomitant other tumors; patients with missing initial laboratory or imaging results; patients aged <18 years; patients with chronic comorbidities, such as autoimmune disorders or hepatic failure, which may influence nutritional or immunological status.

The collected data included demographic information such as age and gender, as well as clinical characteristics including HL pathological subtype, disease stage at diagnosis, presence of B symptoms, and laboratory data (International Prognostic Score [IPS], hemoglobin, leukocyte, neutrophil, lymphocyte, platelets, albumin, C-reactive protein, lactate dehydrogenase, sedimentation rate) and HALP scores at initial diagnosis. The HALP score is determined by the formula: hemoglobin (g/L) × albumin (g/L) × lymphocytes (×10^9^/L)]/platelets (×10^9^/L). Additionally, interim and end-of-treatment responses, follow-up periods, status during follow-up (alive, dead), and survival data (progression-free survival [PFS] and overall survival [OS]) were recorded.

The ABVD regimen (Adriamycin, bleomycin, vinblastine, and dacarbazine) was selected as the initial treatment based on the payment conditions of the national social security institution.^[13]^ PET-CT imaging was employed for disease staging. Patients with liver, spleen, and bone marrow involvement were recorded using PET-CT at the initial diagnosis stage, with bone marrow involvement confirmed via bone marrow biopsy. The response of the patient cohort was assessed using the “PET 5-Point Scale (Deauville Criteria)” in accordance with the Lugano criteria,^[14]^ with those achieving a Deauville score of 1 to 3 classified as responders.

In our study, we aimed to assess the effect of HALP scores, calculated at initial diagnosis, on the clinical and laboratory characteristics of HL at initial diagnosis, as well as on interim and end-of-treatment response and survival outcomes.

Ethics committee approval was obtained (Istanbul Basaksehir Cam and Sakura City Hospital, approval date and number: 28/06/2024-KAEK/26.06.2024.54), and patients and healthy controls gave written informed consent before beginning the study. This study was conducted in accordance with the principles of the Declaration of Helsinki.

## 3. Statistical analysis

In data analysis, the mean, standard deviation, minimum and maximum, and median (25–75% quartile) values were utilized to describe the characteristics, while frequency and percentage values were employed to define categorical variables. The Student *t* test or Mann–Whitney *U* test was utilized to compare the means of 2 independent groups. The Chi-Square test was utilized to assess the relationship between categorical variables. Total survival curves were estimated using the Kaplan–Meier method. Differences based on risk factors were assessed using the log-rank test, and hazard ratios (HR) were reported with 95% confidence intervals (CI). A multivariate Cox hazards regression model was employed to assess prognostic factors influencing the development of progression. The independent contribution of each variable to the event risk is presented with the HR and 95% CI. The model’s discriminatory power was assessed using Harrell’s C-index, and 95% CIs were calculated. The IPS was recorded as a baseline clinical risk indicator. Since IPS was originally developed and validated primarily for advanced-stage HL, it was not used as an independent prognostic variable for the entire cohort. Its interpretation in the overall cohort was considered descriptive, and its clinical relevance was evaluated with caution, particularly because early-stage patients were also included. No imputation was performed for missing data. Analyses were conducted using available cases for each variable. Therefore, percentages for categorical variables were calculated based on the number of patients with available data for the corresponding variable, and variable-specific denominators were reported in the tables.

ROC curve analysis was initially performed to determine an outcome-based cutoff value for the HALP score. Since ROC analysis did not provide a statistically significant threshold, an ROC-derived cutoff was not used. In the absence of a validated clinical threshold for HALP in this study population, the cohort-specific median HALP value was used as a distribution-based cutoff. Median-based categorization is a commonly used approach for dividing continuous variables and biomarkers into low and high groups when no validated clinical cutoff is available.^[15-17]^ Accordingly, patients were divided into low-HALP and high-HALP groups according to the median HALP score of 23.72. The level of statistical significance was set at *P* < .05. Data evaluation was conducted using IBM SPSS Version 25 and the MedCalc statistical software package.

## 4. Results

A total of 100 patients with classical HL were included in the study. The median age of the patients was 33 years (range: 18–82 years), and the median follow-up period was 21 months (range: 1–50). HL was classified as nodular sclerosis in 67% of cases, mixed cellular in 26%, lymphocyte-rich in 6%, and lymphocyte-depleted in 1%. Disease staging showed that 13% of patients were at stage III and 29% at stage IV. At the end of treatment, a complete response (CR) was achieved in 82.8% of cases. During the follow-up period, disease progression was observed in 19% of patients, and mortality was recorded in 10% (Table 1). Patients were categorized based on the median HALP score of 23.72. The mean age was 33.2 ± 13.8 years for patients with a HALP score of <23.72 and 40.42 ± 14.3 years for those with a HALP score of ≥23.72 (*P* = .01). Patients with a low HALP score exhibited significantly higher rates of stage IV disease (44% vs 14%, *P* = .008), presence of B symptoms (76% vs 34%, *P* < .001), and bulky masses (30% vs 6%, *P* = .002). Additionally, patients with a low HALP score had significantly more extranodal involvement compared to those with a high HALP score (46% vs 14%, *P* < .001). Although there was no significant difference between the 2 groups in terms of interim response, patients with a high HALP score demonstrated significantly more CR (91.5% vs 72.5%, *P* = .02) and less progression (10% vs 28%, *P* = .04) at the end of treatment (Table 2). IPS values were presented descriptively to characterize baseline clinical risk. However, because the cohort included both early-stage and advanced-stage patients, IPS was not interpreted as a validated prognostic score for the entire study population.

**Table 1:** Patients’ descriptive characteristics (n = 100).

		Mean ± SD	Median (Min–Max)
Age, (n = 100)		36.8 ± 14.4	33 (18–82)

			%
HL subtypes, (n = 100)	Nodular sclerosis		67
	Mixed cellular		26
	Lymphocyte-rich		6
	Lymphocyte-depleted		1
Stage, (n = 100)	I		7
	II		51
	III		13
	IV		29
B symptom, (n = 100)	(−)		45
	(+)		55
Bulky mass (>8 cm), (n = 100)	(−)		82
	(+)		18
Bone marrow involvement, (n = 12)	(−)		75
	(+)		25
Extranodal involvement, (n = 100)	(−)		70
	(+)		30
Number of involved extranodal sites, (n = 30)	1		66.7
	2		20
	3		10
	4		3.3
Interim response, (n = 95)	CR		67.4
	Others		32.6
End-of-treatment response, (n = 87)	CR		82.8
	Others		17.2
Progression, (n = 100)	(−)		81
	(+)		19
Last status, (n = 100)	Alive		90
	Exitus		10

		Mean ± SD	Median (Min−Max)
Follow-up duration (months), (n = 100)		21.77 ± 10.93	21 (1–50)
HALP Score, (n = 100)		29.79 ± 13.93	23.72 (1.52–99.78)
IPS, (n = 100)		2.18 ± 1.71	2 (0–6)
Hb (g/L), (n = 100)		119.81 ± 23.53	123 (63–173)
Albumin (g/L), (n = 100)		40.69 ± 6.35	42 (22–52)
Lymphocyte (×10^9^/L), (n = 100)		1.73 ± 0.76	1.72 (0.10–4.10)
Platelets (×10^9^/L), (n = 100)		364.61 ± 87.47	355 (104–826)
Neutrophil (×10^9^/L), (n = 100)		6.85 ± 6.04	7.17 (1.05–29.83)
Leukocyte (×10^9^/L)		11.57 ± 6.55	10.04 (1.37–32.87)
C-reactive protein (mg/L)		67.07 ± 80.81	35.3 (0.40–300)
Sedimentation (mm/h)		43.24 ± 23.71	36 (2–140)
LDH (IU/L)		231.78 ± 98.12	201 (100–735)

n values indicate the number of patients with available data for each variable. Percentages were calculated using the variable-specific denominator. Missing data were not imputed.

IPS values were presented descriptively to characterize baseline clinical risk. However, because the cohort included both early-stage and advanced-stage patients, IPS was not interpreted as a validated prognostic score for the entire study population.

HALP = hemoglobin, albumin, lymphocyte, and platelet, Hb = haemoglobin, HL = Hodgkin lymphoma, IPS = International Prognostic Score, LDH = lactate dehydrogenase, SD = standard deviation.

**Table 2:** Assessment of the difference/relationship between HALP score and patients’ characteristics.

		HALP Score		*P*-value
		<23.72 (Low) n = 50	≥23.72 (High) n = 50	
		Mean ± SD	Mean ± SD	
Age		33.2 ± 13.8	40.42 ± 14.3	**.01** *

		n (%)	n (%)	
HL subtypes	Nodular sclerosis	41 (82)	26 (52)	**.001** ††
	Mixed cellular	8 (16)	18 (36)	
	Lymphocyte-rich	**0 (0**)	**6 (12**)	
	Lymphocyte-depleted	1 (2)	0 (0)	
Stage	I	2 (4)	5 (10)	**.008** ††
	II	21 (42)	30 (60)	
	III	5 (10)	8 (16)	
	IV	22 (44)	7 (14)	
B symptom	(-)	12 (24)	33 (66)	**<.001** ††
	(+)	38 (76)	17 (34)	
Bulky mass (>8 cm)	(-)	35 (70)	47 (94)	**.002** ††
	(+)	15 (30)	3 (6)	
Bone marrow involvement	(-)	5 (62.5)	4 (100)	.23††
	(+)	3 (37.5)	0 (0)	
Extranodal involvement	(-)	27 (54)	43 (86)	**<.001** ††
	(+)	23 (46)	7 (14)	
Number of involved extranodal sites	1	15 (65.2)	5 (71.4)	.99††
	2	5 (21.7)	1 (14.3)	
	3	2 (8.7)	1 (14.3)	
	4	1 (4.3)	0 (0)	
Interim response	CR	29 (63)	35 (71.4)	0.51††
	Others	17 (37)	14 (28.6)	
End-of-treatment response	CR	29 (72.5)	43 (91.5)	**.02** ††
	Others	11 (27.5)	4 (8.5)	
Progression	(-)	36 (72)	45 (90)	**.04** ††
	(+)	14 (28)	5 (10)	
Last status	Alive	44 (88)	46 (92)	.50††
	Exitus	6 (12)	4 (8)	

		Mean ± SD Median(Q1-Q3)	Mean ± SD Median(Q1-Q3)	
IPS		2.94 ± 1.64	1.42 ± 0.89	**<.001** *
Hb (g/L)		104.74 ± 20.14	134.92 ± 15.41	**<.001** *
Albumin (g/L)		37.3 ± 5.94	44.02 ± 4.75	**<.001** *
Lymphocyte (×10^9^/L)		1.33 ± 0.66	2.15 ± 0.63	**<.001** *
Platelets (×10^9^/L)		433.15 ± 98.42	296.98 ± 81.54	**<.001** *
Neutrophil (×10^9^/L)		10.58 ± 7.31 8.95 (4.84–13.52)	6.69 ± 3.44 5.92 (4.25–7.89)	**.003** †
Leukocyte (×10^9^/L)		13.23 ± 8.16 11.24 (6.95–16.97)	9.91 ± 3.68 9.27 (7.26–11.24)	.07†
C-reactive protein (mg/L)		102.09 ± 88.35 72.7 (18.65–185.67)	32.18 ± 50.03 12.1 (3.95–47.65)	**<.001** †
Sedimentation (mm/h)		59.48 ± 26.09	26.67 ± 20.85	**<.001** *
LDH (IU/L)		257.32 ± 88.25	205.04 ± 62.89	**.007** *

Bold values indicate *P* < .05, which are considered statistically significant in research.

Data are presented as mean ± standard deviation, median (Q1–Q3), or n (%), as appropriate. Percentages were calculated using the number of patients with available data for each variable. Student *t* test or Mann–Whitney *U* test was used for continuous variables, and the chi-square test was used for categorical variables, as appropriate.

IPS values were presented descriptively to characterize baseline clinical risk. However, because the cohort included both early-stage and advanced-stage patients, IPS was not interpreted as a validated prognostic score for the entire study population.

Q1–Q3: 25% to 75%.

HALP = hemoglobin, albumin, lymphocyte, and platelet, Hb = haemoglobin, HL = Hodgkin lymphoma, IPS = International Prognostic Score, LDH = lactate dehydrogenase, SD = standard deviation.

* Student’s *t*-test.

† Mann–Whitney *U* test.

†† Chi-square test.

Table 2 presents the mean ± standard deviation and median (25–75% quartile) values for the difference assessment, alongside the number (%) values for the association assessment. Patients with a HALP score of <23.72 exhibited a median PFS of 932 days (95% CI: 794–1069) and a 52% probability of non-progression. In contrast, patients with a HALP score of ≥23.72 demonstrated a longer median PFS of 1243 days (95% CI: 1108–1378) and a 79% probability of non-progression. The analysis revealed a statistically significant 2.82-fold (95% CI: 1.14–6.97) increased risk of progression in patients with a HALP score of <23.72 compared to those with a score of ≥23.72 (*P* = .02; Fig. 1). However, statistical analysis concerning OS did not yield significant results. Patients with a HALP score of <23.72 had a median OS of 1157 days (95% CI: 1050–1264) and a survival probability of 87%, whereas those with a HALP score of ≥23.72 had a median survival time of 1310 days (95% CI: 1218–1402) and a survival probability of 91% (*P* = .45; Fig. 2).

**Figure 1.**
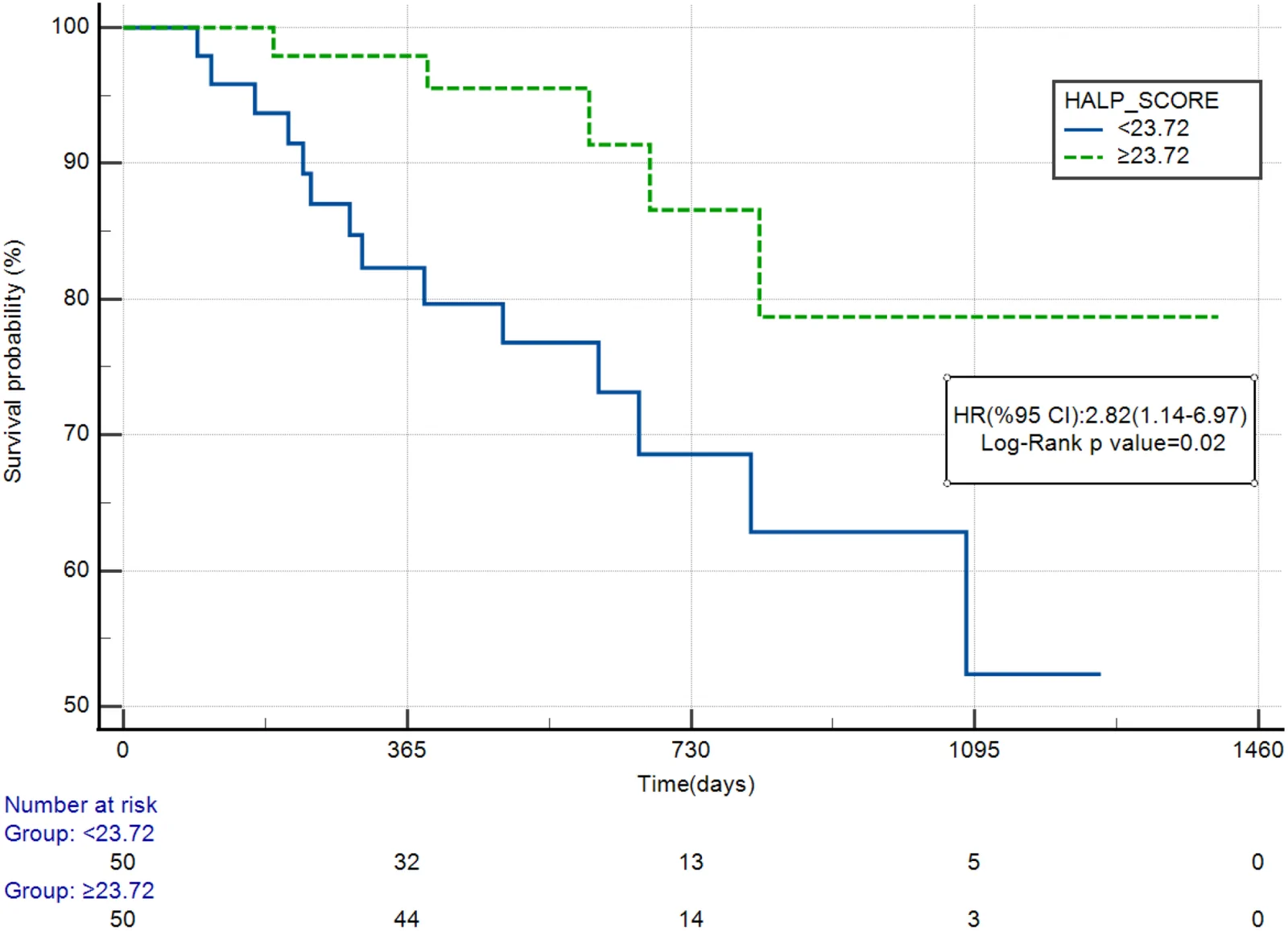
Kaplan–Meier curve for progression-free survival according to HALP score. CI = confidence intervals, HALP = hemoglobin, albumin, lymphocyte, and platelet, HR = hazard ratio.

**Figure 2.**
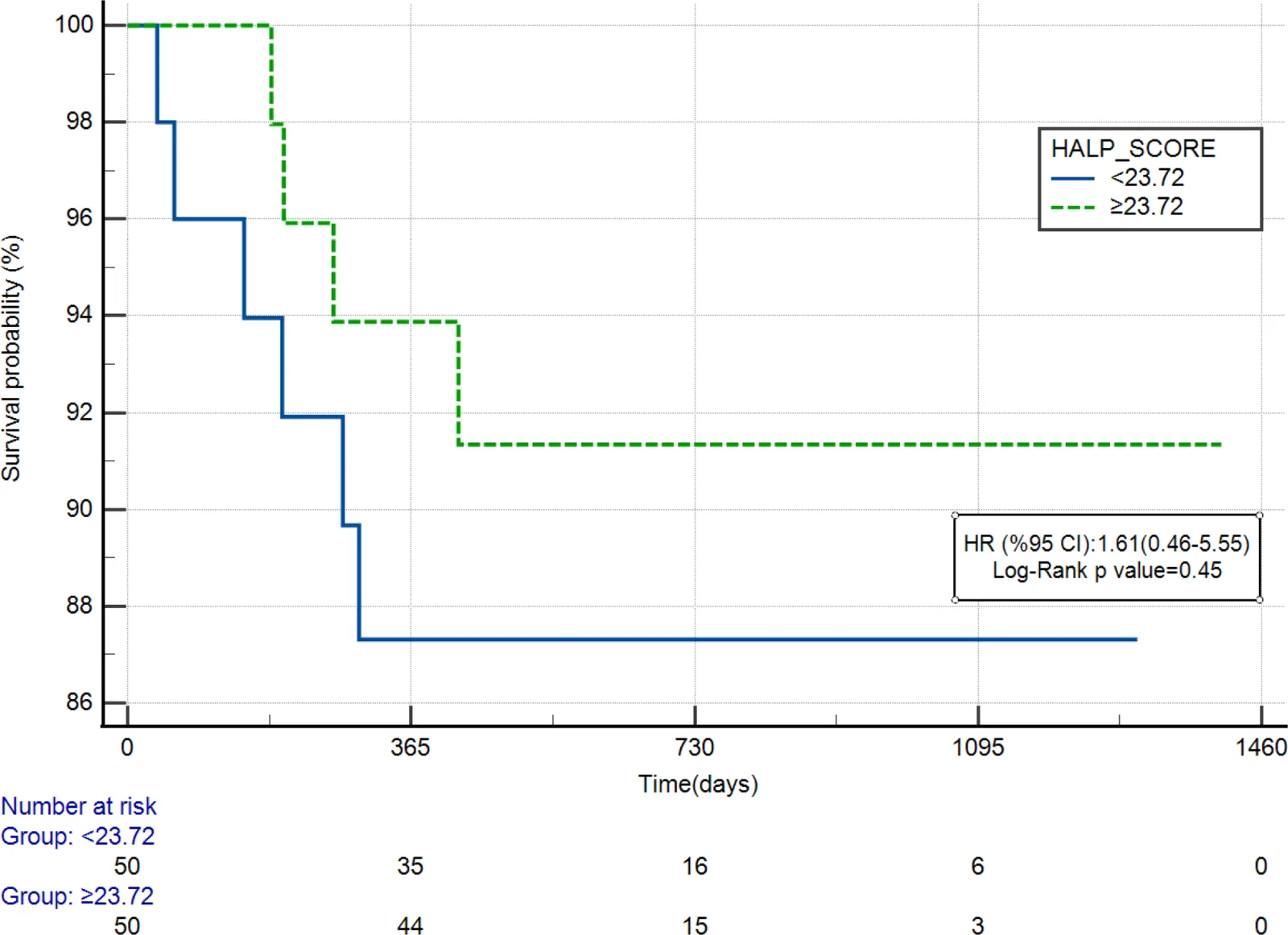
Kaplan–Meier curve for overall survival according to HALP score. CI = confidence intervals, HALP = hemoglobin, albumin, lymphocyte, and platelet, HR = hazard ratio.

Table 3 presents the results of the Cox regression analysis. The Cox regression model demonstrated strong statistical significance, with the variables (age, stage, B symptom, bulky mass, interim response, HALP score) significantly explaining the risk of progression (*P* < .001). The model’s prognostic performance was assessed using Harrell’s C-index, yielding a value of 0.834 (95% CI: 0.766–0.902), indicating a high level of accuracy in distinguishing patients at risk of relapse. Notably, age, stage, and the presence of bulky mass were not statistically significant (*P* > .05 for all). For B symptom (no/yes), the HR was 0.18 (95% CI: 0.05–0.67, *P* = .01), suggesting an 82% reduction in progression risk for those without B symptoms. Regarding interim response (other/CR), the HR was 7.48 (95% CI: 2.52–22.16, *P* = .003), indicating that patients with responses other than CR had a 7.5-fold increased risk of progression. For the HALP score (<23.72/≥23.72), the HR was 2.48 (95% CI: 0.74–8.31, *P* = .14); patients with a HALP score of <23.72 exhibited a 2.5-fold increase in progression risk, although this finding was not statistically significant. Figure 3 presents a curve derived from the Cox regression model, which accounts for the influence of variables such as age, stage, B symptom, bulky mass, interim response, and HALP score. The curve indicates that the PFS duration did not fall below 50%, suggesting a generally favorable prognosis for the cohort. The 4-year PFS rate was approximately 74%. Clinically, these findings indicate that patients with low HALP scores had shorter PFS than those with high HALP scores, and that this progression-free survival disadvantage persisted throughout the follow-up period. However, the precision of late survival estimates is lower due to the limited number of events and the decreasing number of patients at risk during longer follow-up.

**Table 3:** Cox regression analysis.

Covariates	HR (%95 CI)	*P*-value
Age	0.99 (0.99–1.03)	.61
Stage (III-IV/I-II)	0.68 (0.23–2.01)	.49
B symptom (no/yes)	0.18 (0.05–0.67)	**.01**
Bulky mass (>8 cm; yes/no)	0.41 (0.12–1.37)	.14
Interim response (other/CR)	7.48 (2.52–22.16)	**.003**
HALP score (<23.72/≥23.72)	2.48 (0.74–8.31)	.14

Bold values indicate *P* < .05, which are considered statistically significant in research.

CR = complete response, HALP = hemoglobin, albumin, lymphocyte, and platelet.

**Figure 3.**
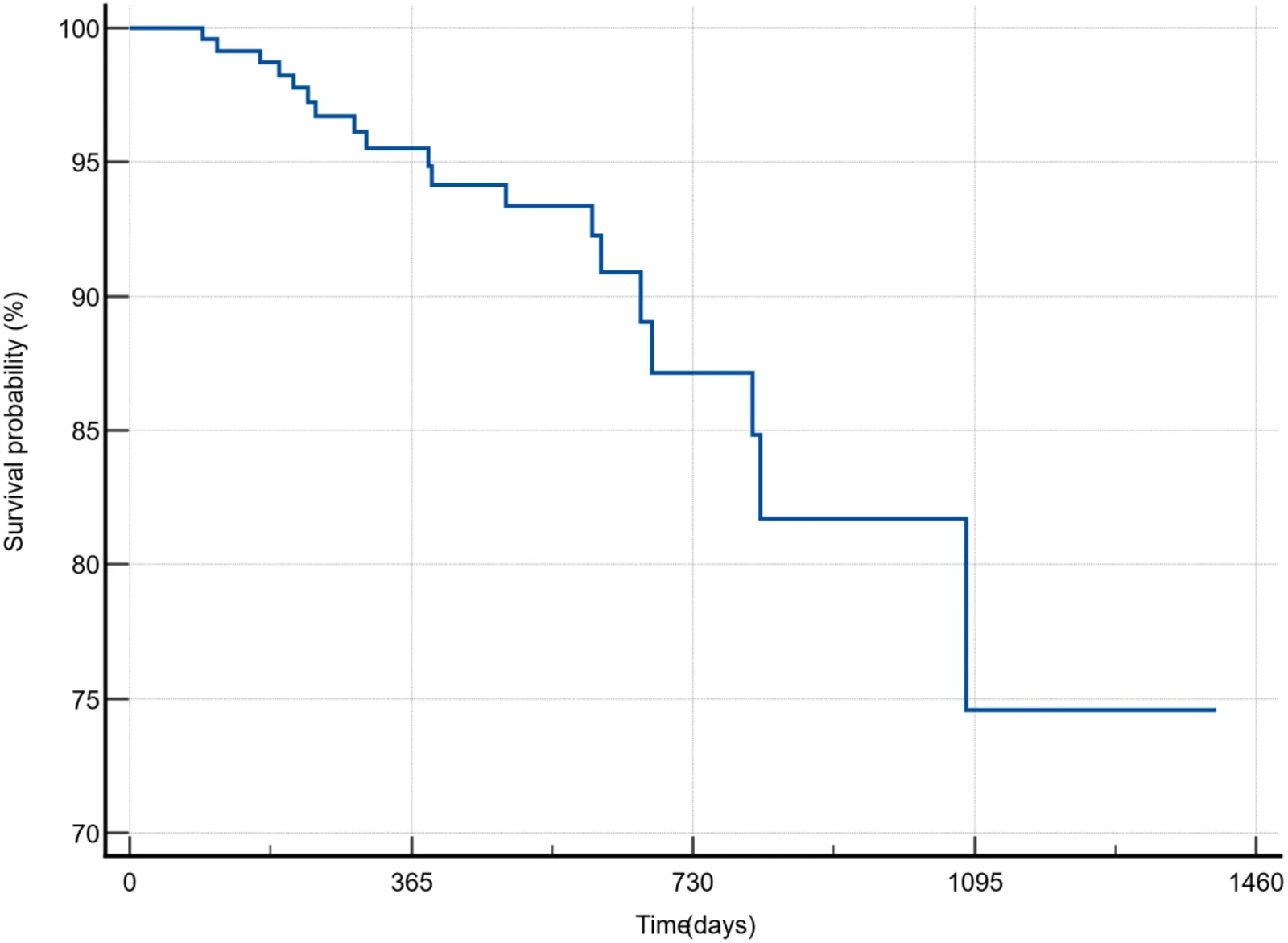
Adjusted survival curve derived from the Cox regression model.

## 5. Discussion

This study has demonstrated significant results concerning the predictive value of the HALP score at initial diagnosis in patients with HL for both treatment response and survival outcomes. A review of the literature indicates a predominant focus on the association between the HALP score and solid malignancies. The findings of our study will offer significant insights for future investigations in this field.

A study from 2025 involving 147 HL patients, which closely resembled our cohort, examined additional predictors such as neutrophil-lymphocyte ratio, lymphocyte-monocyte ratio, and platelet-lymphocyte ratio at diagnosis concerning disease characteristics and survival. This study found that a high HALP score correlated with early-stage favorable disease (*P* = .034), though no significant differences in survival outcomes were reported.^[18]^ Another study of 1407 diffuse large B-cell lymphoma patients assessed the prognostic implications of the HALP score alongside age, Eastern Cooperative Oncology Group performance scores, extranodal involvement, lactate dehydrogenase, advanced stage, and low albumin levels, as well as the neutrophil-lymphocyte ratio.^[12]^ It was determined that a low HALP score (≤13, AUC 0.58; 95% CI 0.56 to 0.61; *P* < .001) served as a poor prognostic indicator associated with reduced 3-year survival.^[12]^ Our study identified numerous characteristics linked to low HALP scores, which were significantly associated with high-risk factors: Stage IV disease, B symptoms, bulky masses, and extranodal involvement. Patients with higher HALP scores achieved higher end-of-treatment CR rates compared to those with lower scores and, as a novel finding in the literature, exhibited significantly prolonged PFS.

In a 2024 study, the efficacy of the HALP score was assessed in 74 patients with testicular tumors, revealing that the HALP score was significantly lower in metastatic patients, as well as those with progression and mortality, compared to the other group.^[19]^ In another study conducted in the same year involving 2053 patients with non-small cell lung cancer, it was demonstrated that patients with a high HALP score had longer 5-year OS compared to those with a low HALP score.^[20]^ In patients with colorectal cancer, a high HALP score was shown to be an independent poor prognostic factor for both disease-free survival and OS.^[21]^ In patients with gastric cancer, another gastrointestinal tumor, a high HALP score is a significant prognostic factor for OS.^[22]^ A meta-analysis from 2024 systematically examined the predictive value of HALP for survival outcomes in patients with solid tumors.^[23]^ A high HALP score was significantly associated with favorable OS, cancer-specific survival, PFS, and disease-free survival.^[23]^ The hypothesis posited that the HALP score and its associated parameters could also prove effective in the progression of HL. The results of the present study appear to substantiate this hypothesis. In our study, we clearly demonstrated the association between adverse tumoral characteristics and a low HALP score in patients with HL. Furthermore, we demonstrated that a high HALP score serves as a guide for improved treatment response and prolonged PFS. Patients with a low HALP score exhibited significantly higher rates of stage IV disease, presence of B symptoms, bulky masses, and extranodal involvement compared to those with a high HALP score. Patients with a high HALP score showed significantly more CR and less progression at the end of treatment. Our results also revealed a statistically significant 2.82-fold increased risk of progression in patients with a HALP score of <23.72 compared to those with a score of ≥23.72. No significant result was obtained in terms of OS. The HR identified in the Cox regression analysis is clinically significant concerning the HALP score. Patients with a HALP score of <23.72 demonstrated a 2.5-fold increase in the risk of progression, although this result did not achieve statistical significance. Although a low HALP score was significantly associated with shorter PFS in univariate analysis, it did not retain statistical significance in the multivariate Cox regression model after adjustment for established clinical factors. This finding suggests that the prognostic association of HALP may partly reflect disease burden rather than independent prognostic biology. In the present cohort, lower HALP scores were also associated with advanced-stage disease, B symptoms, bulky mass, extranodal involvement, and higher inflammatory markers. Therefore, HALP may represent a composite indicator of tumor burden, inflammation, and nutritional/immune status rather than an independent determinant of disease progression.

Programmed death receptor-1 blockade is recognized as a safe and effective therapeutic approach for relapsed/refractory classical HL.^[24-27]^ Nivolumab, a programmed death receptor-1 inhibitor, is widely utilized as an immune checkpoint inhibitor and is favored in the treatment of HL across various stages.^[6,24-27]^ A 2025 study investigated the relationship between the HALP score and the efficacy of immune checkpoint inhibitors in patients diagnosed with metastatic renal cell carcinoma who received nivolumab treatment. The analysis of the patient data revealed that patients with a low HALP score exhibited shorter PFS and OS.^[28]^ Another study identified a significant association between a lower HALP score and reduced OS in patients with advanced cancer receiving immune checkpoint inhibitors.^[29]^ Although our study did not include patients treated with immune checkpoint inhibitors, consistent with current literature, it can be suggested that calculating the HALP score prior to the administration of these agents – expected to be preferred in early treatment stages in the future – will be essential for predicting survival and treatment response.

There were some limitations in our study. The most significant limitation is that it includes data from a single center. For this reason, our patient sample was limited; in particular, HL subtype analyses could not be performed. The relatively short follow-up period was also another limitation of our study. The limited duration of follow-up may have influenced the results of the survival analysis, potentially contributing to the absence of significant findings in OS, despite the observed significance in PFS. It is important to consider that extending the follow-up period and increasing the patient cohort size could address this limitation. Although stage-specific evaluation of HALP may provide additional clinical insight, the limited number of progression events in the present cohort restricted reliable subgroup analysis. Further stratification into early-stage and advanced-stage subgroups would have resulted in very small event numbers, making stage-specific multivariate Cox regression statistically unstable and prone to overfitting. Therefore, the stage-specific prognostic value of HALP should be investigated in larger cohorts with sufficient event numbers. The most significant strength of our study is that highly consistent results could be obtained with complete patient data.

In conclusion, lower HALP scores were associated with adverse disease characteristics, poorer end-of-treatment response, and shorter PFS in patients with HL. However, HALP did not remain an independent predictor of progression in the multivariate Cox regression model. Therefore, HALP may be considered a useful, easily accessible supportive marker reflecting disease burden and systemic inflammatory/nutritional status, rather than an independent prognostic factor. Further prospective studies with larger cohorts are needed to clarify its prognostic role in HL.

## Author contributions

**Conceptualization:** Mesut Ayer.

**Data curation:** Mesut Ayer, Gunhan Tekin, Edibe Sevde Eker, Istemi Serin.

**Formal analysis:** Mesut Ayer, Istemi Serin.

**Methodology:** Mesut Ayer.

**Supervision:** Mesut Ayer.

**Writing – original draft:** Mesut Ayer, Gunhan Tekin, Edibe Sevde Eker, Istemi Serin.

**Writing – review & editing:** Mesut Ayer, Gunhan Tekin, Edibe Sevde Eker, Istemi Serin.
